# Prevalence and Risk Factors of Asthma in Children and Adolescents in Rabigh, Western Saudi Arabia

**DOI:** 10.3390/children10020247

**Published:** 2023-01-30

**Authors:** Turki S. Alahmadi, Moustafa A. Hegazi, Hani Alsaedi, Hanaa Hamadallah, Ali F. Atwah, Abdulrahman A. Alghamdi, Haya M. Altherwi, Majd S. Alghamdi, Ebtihal M. Albeshri, Moud I. Alzanbaqi, Abubaker M. Bamakhish, Mohamed S. El-Baz

**Affiliations:** 1 Department of Pediatrics, Faculty of Medicine, King Abdulaziz University, Jeddah 21589, Saudi Arabia; 2 Department of Pediatrics, Faculty of Medicine in Rabigh, King Abdulaziz University, Jeddah 80205, Saudi Arabia; 3 Department of Pediatrics, Mansoura University Children’s Hospital, Mansoura 35516, Egypt; 4 Faculty of Medicine in Rabigh, King Abdulaziz University, Jeddah 80205, Saudi Arabia; 5 Department of Pediatrics, Faculty of Medicine, Cairo University, Cairo 11562, Egypt

**Keywords:** asthma, air quality, prevalence, risk factors, children and adolescents, Rabigh, Saudi Arabia

## Abstract

The worldwide prevalence of asthma in children is variable. The different epidemiological definitions of asthma, the use of various methods of measurement, and the environmental variations between countries are responsible for such different prevalence rates. This study has been performed to identify the prevalence/risk factors of asthma in Saudi children/adolescents in Rabigh. A cross-sectional epidemiological survey has been conducted using the validated Arabic version of the “International Study of Asthma and Allergies in Childhood questionnaire”. Data on the sociodemographic characteristics of participants and risk factors of asthma have also been collected. Three hundred and forty-nine Children/adolescents with an age range of 5–18 years have been randomly selected for an interview from public places and houses in different regions of Rabigh City. The prevalence rates of physician-diagnosed asthma, any wheezing, and wheezing in the last 12 months among children/adolescents (mean age: 12.22 ± 4.14 years) have remarkably increased in association with the rapidly developing industrialization of Rabigh from previously recorded rates of 4.9%, 7.4%, and 6.4% in the only study that has previously been conducted in Rabigh in 1998 to 31.5%, 23.5%, and 14.9%, respectively. The univariate analysis has detected some significant risk factors for asthma. However, in younger aged children (5–9 years), allergic rhinitis, associated chronic illnesses, and viral respiratory infection-induced wheezing have remained significant risk factors of any wheezing. Drug allergy, exposure to dust, and viral respiratory infection-induced wheezing have persisted as significant risk factors for wheezing in the last 12 months. Eczema in the family, exposure to perfumes/incense, and viral respiratory infection-induced wheezing have remained as significant risk factors of physician-diagnosed asthma. The results of this survey should be useful in future targeted preventive plans/measures with special attention to improving air quality to limit the progressive increase in asthma prevalence in Rabigh, as well as in other similar industrial communities.

## 1. Introduction

Asthma is the most widespread persistent and recurring illness in children and young adults and represents a major public health problem with many serious impacts, including frequent morbidities/disabilities, a high burden on healthcare resources, and poor quality of life for asthmatic patients with loss of their work productivity, including absenteeism from school for children and work for adults [1,2].

The worldwide prevalence of asthma is variable with a range of 1–20% in both children and adults. The different epidemiological definitions of asthma, the use of various methods of measurement, and the environmental variations between countries are responsible for such different prevalence rates [3].

In Saudi Arabia, a unique country that has moved from a primarily rural to a wealthy urban economy over the past five decades with diverse topographic features and climate conditions, prevalence rates of allergic disorders including asthma are expected to be high in association with industrialization, or westernization, and change of population lifestyle as has occurred in developed countries. The highest prevalence rates of asthma in Saudi children have been found in Hofuf (33.7%), Najran (27.5%), and Madinah (23.6%), while the lowest prevalence rates have been found in Abha (9%), which is a rural southern mountain region, Qassim (3.2%), which is an oasis-like northern rural region famous for agricultural farms, and Dammam (3.6%). The high rate of asthma prevalence is related to some risk factors including socioeconomic status, the spread of urbanization, dietary habits, lifestyle changes, increased exposure to tobacco smoke, sandstorms, dust, and vehicular/industrial pollutants [4,5,6].

In studies conducted over the past 3 decades in Saudi children, the overall prevalence of asthma has ranged from 8–25% [5]. In a recent review, the prevalence of childhood asthma has been varied among different regions of Saudi Arabia. Male gender, having pets at home, dietary habits, and exposure to environmental factors have been associated with a higher prevalence of asthma [6].

In our locality, Rabigh, a growing industrialized city in the western region of Saudi Arabia on the red seacoast 180 km to the north of Jeddah city with an estimated population of 180,000, very limited or insufficient data about the prevalence/risk factors of asthma, whether in children or adults, are available. Only one study, which has been conducted 24 years ago in Rabigh, has detected that the prevalence rates of physician-diagnosed asthma and any lifetime wheezing in middle school children are 4.9% and 7.4%, respectively [7].

The prevalence of asthma/asthma symptoms in Rabigh is expected to currently be higher due to significantly and rapidly developing industrialization. Many factories have been established in the past 2 decades around Rabigh City, mainly distributed in the western and southern regions of the city, and they include petroleum, cables, cement, and other heavy industries. Also, there are other environmental factors related to non-industrial air pollution, considering traffic-related air pollution and the frequently encountered sandstorms from the eastern desert side of the city.

There is a need for a periodical evaluation of the local prevalence/risk factors of asthma to adapt an appropriate and targeted control and prevention strategy. Therefore, the current study has been performed to identify the prevalence/risk factors of asthma in Saudi children and adolescents in the industrial city of Rabigh in the western region of the Kingdom of Saudi Arabia (KSA).

## 2. Material and Methods

### 2.1. Study Design and Main Measurement Tool

A cross-sectional epidemiological survey has been conducted utilizing the validated Arabic version of the “*International Study of Asthma and Allergies in Childhood (ISAAC) questionnaire*” for the prevalence of asthma/asthma symptoms in children/adolescents. The worldwide multicenter ISAAC study has been conducted in many countries and has offered the initial assessment of the prevalence/severity of asthma among children of different populations [8]. Children/adolescents with the age range of 5–18 years have been randomly selected and conveniently recruited for a face-to-face interview in public places (gardens, recreation areas, shopping malls, and streets), as well as from houses in different regions of Rabigh City. The study has been conducted from June 2021 to June 2022 after the end of lockdown measures in Saudi Arabia and a marked decrease in the number of COVID-19 cases to facilitate interviews, which have been done to accurately collect participants’ responses.

### 2.2. Exclusion Criteria

Young children less than 5 years old have been excluded to avoid the difficulties associated with a confirmed diagnosis of asthma and the well-known high prevalence of transient early wheezing with respiratory infections/colds in young children. Children with respiratory disorders associated with asthma-like symptoms (e.g., cystic fibrosis and obstructions of the large or small airways of variable causes), children with a history of prematurity/low birth weight and bronchopulmonary dysplasia, and non-Saudi children as well questionnaires without completely filled data have been excluded from this study.

### 2.3. Measurement Tools/Implementation

In addition to the validated ISAAC questionnaire for identifying the prevalence of asthma/asthma symptoms in children/adolescents, another two sections have been included: one section for the sociodemographic characters of the participants, and the other section for almost all possible risk factors of asthma, including various forms of atopy in the recruited children/adolescents and their parents, indoor allergens (e.g., pets at home such as cats or birds, moquette/carpets in rooms, exposure to passive smoking, perfumes/incense, and plants), outdoor allergens (e.g., exposure to dust/sandstorms), and air pollution (i.e., living near main roads or industrial areas). The risk factors section of the questionnaire also includes data about parental consanguinity, mode of delivery, gestational age, exclusive breastfeeding duration, associated chronic illnesses, including obesity, and dietary habits, especially daily vegetables/fruits intake and frequent consumption of fast foods more than twice/week.

The whole questionnaire has been self-completed by 8–18-year-old children/adolescents who could understand the questions with or without the help of their parents or the interviewer. A parental-reported/completed questionnaire has been applied for 5–8-year-old children who could not understand or provide the required responses without the help of their parents or the interviewer.

The interviews have been conducted by medical students after being trained on the method of the study and the skills of the interview.

### 2.4. Definitions of Asthma/Asthma Symptoms

In this study, asthma has been defined as physician-diagnosed asthma, which is based on the question: “*Have you ever had asthma or been told by a physician to have asthma?*”

The asthma symptoms have been based on responses to: “Did you have wheezing without colds in the last 12 months?” “Did you have wheezing during or after exercise in the last 12 months?” “Did you have a dry cough at night, apart from a cough associated with a cold or a chest infection in the last 12 months?”

### 2.5. Sample Size/Study Power

The sample size has been calculated according to the published data on the highest prevalence of asthma/asthma symptoms in 34% of Saudi children/adolescents [4,6] and utilizing the same measuring tool (ISAAC questionnaire) [8].

An estimated sample size of 245 children/adolescents (5–18 years) in Rabigh has achieved 90% study power to reveal a 10% difference between the hypothesized proportion of 34% and the alternative hypothesis that the maximum expected proportion is 44% using a two-sided, binomial hypothesis test with type I error of 5%. However, 349 complete responses could be collected, which exceeds the required estimated sample size and extremely increases the power of the study to more than 95%. There are only 33 incomplete responses and the rate of refusal to participate is 15%.

### 2.6. Ethical Approval

The biomedical ethics unit of the Faculty of Medicine of King Abdulaziz University has issued the approval for this study (Reference No 561-20-20), approval date: 13 August 2020.

### 2.7. Informed Consent

The parents of participating children/adolescents have provided the required written informed consent. The consent for participation in this survey has also been considered whenever the questionnaire is filled out by participants/their parents, in compliance with the Declaration of Helsinki.

### 2.8. Statistical Analysis

Data have been analyzed by IBM’s Statistical Package for the Social Sciences (SPSS) after a thorough check to exclude any incomplete or inconsistent data. Data have been double-checked and scrutinized before and after entry into the SPSS program. Categorical variables have been presented as proportions, whereas non-categorical variables have been presented as means and standard deviations (SD). Qualitative data between children/adolescents with and without asthma/asthma symptoms have been compared by Chi-square test. Univariate logistic regression with the calculation of unadjusted odds ratios (ORs) and 95% confidence interval (CI) has been done to determine the association between each variable and the outcome variable (asthma/asthma symptoms). Significant variables (risk factors) in univariate analyses are included in multivariate logistic regressions to adjust for confounders, calculate the adjusted ORs (AORs), and detect the most significant risk factors of asthma/asthma symptoms in children/adolescents. Significance is considered at *p*-value < 0.05.

## 3. Results

Three hundred and forty-nine children and adolescents living in Rabigh City fully completed the three sections of the questionnaire. There are 144 males (41.3%) and 205 females (58.7%) (mean and SD of age: 12.22 ± 4.14 years and age range from 5–18 years) without significant differences in the gender or age of participants. The age and gender of participants are representative of the children and adolescent population in Rabigh. The eastern region of Rabigh City has the highest proportion of participants (32.4%), followed by the central region, which has 19.5% of participating children/adolescents. Eighty-two participants (23.5%) are living near factories with forty-one of them (11.7%) live near to Petro Rabigh. Ninety-five participants (27.2%) are living near main roads. Living in a rented apartment is the most common type of home for 36.4% of participants. A monthly family income of more than 10,000 SAR is the most common income for 38.4% of participants’ families. University level of education is the main education level for 42.4% of fathers and 53.3% of mothers of the participants, and 78.2% of fathers and 63.9% of mothers are employed full-time. Table 1 presents the sociodemographic characteristics of participants.

The prevalence of physician-diagnosed asthma, any wheezing and wheezing in the past 12 months in children/adolescents in Rabigh City are 31.5%, 23.5%, and 14.9%, respectively. Forty-three children/adolescents out of 52 (82.7%) have had 1–3 instances of wheezing attacks, 34 children/adolescents out of 52 (65.4%) have had awakening/disturbed sleep due to wheezing, and 31 children/adolescents out of 52 (59.6%) have had a severe wheezing-limited speech in the past 12 months. The prevalence of asthma/asthma symptoms in children/adolescents in Rabigh City according to ISAAC are presented in Table 2.

Univariate analysis of all the studied demographic, socioeconomic, and biomedical variables has identified some significant risk factors of any wheezing, wheezing in the past 12 months, and physician-diagnosed asthma, including residence near main roads, atopic diseases in children/adolescents (i.e., allergic rhinitis, allergic conjunctivitis, drug allergy), atopic diseases in the family, moquette/carpet in rooms, exposure to passive smoking, exposure to burned coal/wood, exposure to perfumes/incense, exposure to dust, viral respiratory infection-induced wheezing, fast food more than once/week, and associated chronic illnesses. Details of the univariate analysis of significant variables associated with any wheezing are provided as Supplementary Material (Table S1).

However, multivariate regression analysis shows that in children 5–9 years (AOR: 0.48, 95% CI: 0.24–0.95, *p* = 0.04), allergic rhinitis in children/adolescents (AOR: 4.22, 95% CI: 1.17–15.17, *p* = 0.03), associated chronic illnesses (AOR: 5.52, 95% CI: 1.59–19.16, *p* = 0.007), and viral respiratory infection-induced wheezing (AOR: 6.64, 95% CI: 2.78–15.89, *p* < 0.0001) are the only significant independent risk factors of any wheezing in children/adolescents in Rabigh city (Table 3). Associated chronic illnesses have been found in 18 children/adolescents, including mainly diabetes mellitus in 7 and psoriasis in 4 children/adolescents.

Multivariate regression analysis reveals that drug allergy in child/adolescents (AOR: 11.34, 95% CI: 1.66–77.48, *p* = 0.01), exposure to dust (AOR: 3.82, 95% CI: 1.10–13.24, *p* = 0.03), and viral respiratory infection-induced wheezing (AOR: 4.63, 95% CI: 1.33–16.05, *p* = 0.01) are the most significant independent risk factors of wheezing in the preceding 12 months in children/adolescents in Rabigh City (Table 4). Details of univariate analysis of significant variables associated with wheezing in the last 12 months are provided as Supplementary Material (Table S2).

Multivariate regression analysis reveals that eczema in the family (AOR: 2.85, 95% CI: 1.10–7.41, *p* = 0.03), exposure to perfumes/incense (AOR: 5.59, 95% CI: 1.95–16.12, *p* = 0.001), and viral respiratory infection-induced wheezing (AOR: 6.35, 95% CI: 2.24–18.02, *p* = 0.001) are the most significant independent risk factors of physician-diagnosed asthma in children/adolescents in Rabigh City (Table 5). Details of univariate analysis of significant variables associated with physician-diagnosed asthma in children and adolescents are provided as Supplementary Material (Table S3).

## 4. Discussion

In this cross-sectional epidemiological survey of asthma/asthma symptoms in Rabigh City, the rates of prevalence of physician-diagnosed asthma, any wheezing, and wheezing in the last 12 months among children/adolescents with a mean age of 12.22 ± 4.14 years are 31.5%, 23.5%, and 14.9%, respectively. The prevalence rates of physician-diagnosed asthma, any lifetime wheezing, and wheezing in the last 12 months in 408 Saudi middle school children with closely similar ages are 4.9%, 7.4%, and 6.4%, respectively, in the only study that has been conducted in Rabigh in 1998 and utilizing the same ISAAC questionnaire [7].

Thus, there is a remarkable and alarming increase in the prevalence of asthma/asthma symptoms in the past 20 years in children/adolescents in Rabigh City. Rabigh is considered to have the second highest prevalence of asthma in Saudi children, after Hofuf in the eastern region of KSA, with a prevalence of 33.7%, recorded by a standardized questionnaire similar to ISAAC [6,9]. Also, the prevalence of physician-diagnosed asthma in children/adolescents in Rabigh is higher than the reported prevalence of physician-diagnosed asthma of 19.6%, but lower than the reported prevalence of lifetime wheezing (25.3%) and wheezing during the last 12 months (18.5%) in adolescents 16 to 18-years-old in a study from Riyadh, which has similarly utilized the ISAAC questionnaire [10] and is higher than the reported general asthma prevalence among Saudi children, from 8% to 25% [6,11]. However, the prevalence of wheezing in the last 12 months of 14.9% recorded in this study is closely similar to the average global prevalence of wheezing in the preceding 12 months, being 14.1% in adolescents 13–14-years-old according to data from ISAAC Phase III. The global prevalence of wheezing in the last 12 months ranges from <5.0% in the Indian subcontinent, Asia-Pacific, Eastern Mediterranean, and Eastern Europe to ≥20% in English language-speaking countries of the UK, Australia, Europe, North America, and parts of Latin America [12,13]. Over the 27-year period (1993–2020), the prevalence of current wheezing has decreased in low-income countries (−1.37, −2.47 to −0.27) in children and (−1.67, −2.70 to −0.64) in adolescents, and increased in lower-middle-income countries (1.99, 0.33 to 3.66) in children and (1.69, 0.13 to 3.25) in adolescents, but it is stable in upper-middle-income and high-income countries. However, the worldwide burden of severe asthma symptoms would be mitigated by enabling access to effective therapies for asthma [14]. This runs in concordance with the findings in the current study, indicating the higher prevalence of asthma in high-income countries than in low-income countries because the higher prevalence is most probably linked to increased industrialization in such higher-income countries.

The remarkable and alarming rise of childhood asthma/asthma symptoms in Rabigh from the previously recorded levels in 1998 is highly consistent with epidemiological studies in KSA, showing that asthma prevalence has been rising in the past 30 years. This is possibly due to a shift of KSA from a primarily rural to a wealthy urban economy with fast alterations related to the industrialization and modification of population life style, as has occurred in high-income developed countries, as well as alterations in dietary habits and an excess of the environmental triggers of asthma exposure, such as indoor allergens and outdoor pollutants [4,15].

Similarly, the prevalence of asthma/asthma symptoms in Rabigh is expected to be currently increasing in association with the significantly and rapidly developing industrialization, with many factories that have been established in the past 2 decades in Rabigh. The 24-h fine particulate matter (PM2.5) indicative of air pollution of 37 ± 16.2 μg m^−3^ in Rabigh significantly exceeds the World Health Organization (WHO) guideline of 20 μg m^−3^, which is alarming when worrying about poor air quality in Rabigh [16]. Additionally, a study shows that the risk for cardiac and respiratory illnesses is increased in response to fine particulate air pollution exposure in Rabigh City [17]. Additionally, the rise in the prevalence of asthma can be explained by the growing awareness of asthma among healthcare providers and the general public, so more individuals are diagnosed with asthma.

Other well-known gene-environmental risk factors of asthma can exert additional important roles in increasing the prevalence/severity of asthma in the Rabigh community. However, the considerable increase in the numbers of children/adolescents with asthma around the world as well as in Rabigh is more possibly related to environmental factors rather than genetic factors, but the method and outcome of the interaction of the factors with each other and with genes are still not known. Globally, ISAAC phase I identifies the same major differences in the prevalence of asthma symptoms, with a diversity of 20–60-fold between centers, displaying remarkable variations in asthma prevalence in populations with similar ethnic or genetic backgrounds [18], supporting that asthma prevalence in a community is mainly determined by environmental risk factors [19]. ISAAC phase III has investigated asthma risk factors and the relations with many environmental risk factors have been identified [20].

The higher childhood asthma/asthma symptoms prevalence in metropolitan areas of industrialized countries and an industrial city like Rabigh, detected in this survey, as well as in Saudi children in the industrial city of Yanbu [21] compared to non-industrial rural communities points out the role of air pollution in asthma evolution [22]. Traffic-related air pollution (TRAP) has been associated with increased rates of hospitalization related to exacerbations of asthma and reduction of pulmonary function [23]. Furthermore, increased exposure to black carbon and PM2.5 during childhood is associated with an increased risk of asthma at age 12, evidenced by meta-analyses of birth cohort studies [24].

In this survey, the role of environmental risk factors has been investigated and only residence near main roads with an increased possibility of TRAP is significantly associated with any lifetime wheezing in univariate analysis, but this significance disappears in multivariate regression analysis. The role of residence near factories and the type of factory could not be proven to be a determinant factor of childhood asthma/asthma symptoms. This could be explained by the presence of factories all around Rabigh City, especially in the southern area, and the important consideration that the exposure of residents in Rabigh to industrial-related air pollution is not mainly dependent on the source of pollution or site of the factory as a major determinant risk factor for childhood asthma development, but is largely dependent on the direction and speed of winds that can carry polluted air to different regions of Rabigh City, as demonstrated in previous studies which have found poor air quality in Rabigh City [16,17]. One of these studies has shown that industrial/vehicular emissions and fossil-fuel combustion significantly contribute to the overall poor air quality in Rabigh [16].

In this study, all the demographic, socioeconomic, and biomedical variables have been investigated and analyzed as possible risk factors associated with childhood asthma development. The univariate analysis detects some significant risk factors of any wheezing, wheezing in the past 12 months, and physician-diagnosed asthma, including residence near main roads, atopic diseases in children/adolescents or their family members, exposure to indoor allergens (moquette/carpet in rooms, passive smoking, burned coal/wood, perfumes/incense) exposure to outdoor allergens (dust, sandstorms), viral respiratory infection-induced wheeze, fast food more than twice/week, and associated chronic illnesses.

However, multivariate regression analysis has shown that in children 5–9 years old, allergic rhinitis, associated chronic illnesses, and viral respiratory infection-induced wheezing are the only significant independent risk factors of any wheezing in children/adolescents in Rabigh City (Table 3). Drug allergy in children/adolescents, exposure to dust, and viral respiratory infection-induced wheezing are the most significant independent risk factors of wheezing in the last 12 months in children/adolescents in Rabigh City (Table 4). Eczema in the family, exposure to perfumes/incense, and viral respiratory infection induced-wheezing are the most significant independent risk factors of physician-diagnosed asthma in children/adolescents in Rabigh City (Table 5). Similarly, these risk factors of childhood asthma have been identified in previous global and national studies [4,5,6,9,10,12,13,14,15,25,26,27,28]. According to WHO, the most powerful risk factors for developing asthma are inhaled particles and substances that may irritate the airways or induce allergic reactions [29].

The younger age could have been significantly associated with any wheezing, mostly due to more prevalent respiratory viral infections-induced wheezing in younger children than adolescents and adults. Regarding allergic rhinitis, the association of rhinitis with asthma symptoms in children is thought to be part of the united airway disease’s theory, which proposes three possible explanatory pathophysiological mechanisms: postnasal dripping, naso-bronchial reflex, and systemic immune response. Anatomical, histological, epidemiologic, pathophysiologic, emerging biomarkers, clinical, and treatment evidences reveal the link between the upper and lower airways, highlighting the strong interaction between allergic rhinitis and asthma. The understanding of the relationship between the upper and lower airways and its contribution to T helper 2 (Th2)-skewed diseases, such as allergic rhinitis with asthma, has led to a novel therapeutic strategy for a comprehensive approach to the treatment of upper airway inflammation with asthma [30,31,32,33,34].

In this study, the most significant and the only risk factor associated with any wheezing, wheezing in the last 12 months, and physician-diagnosed asthma is a viral respiratory infection-induced wheeze. In early life, respiratory virus infections have a critical role in asthma evolution. Particularly, respiratory syncytial virus (RSV) and human rhinovirus (HRV) are significantly correlated with wheezing attacks in young children and with the development of asthma in the following years. Nearly 50% of the infants with RSV infections in the first year of life are noted to have had permanent asthma at school age [34]. In children at high risk for asthma, HRV-induced wheezing during infancy has proven to be the most powerful predictor of physician-diagnosed asthma at 6 years of age [35]. Moreover, other studies have supported this finding, and many viruses that induce wheezing are an important risk factor for developing asthma, and commonly trigger asthma exacerbation in children [36,37]. However, in this study, there is no evidence, published data, or statements by participants that a particular virus, for example, RSV and HRV, is the primary culprit in Rabigh. Additionally, the determination or identification of respiratory viral infections which have major contribution to wheezing requires and deserves another study especially designed for this purpose.

The current cross-sectional epidemiological survey has many strengthening points and unique features, as it is one of the comprehensive community-based studies offering thorough knowledge and recent data about the prevalence/risk factors of asthma/asthma symptoms in children/adolescents in our urban locality, Rabigh City. The inclusion of almost all possible risk factors for bronchial asthma in Saudi children/adolescents, the unique study design, proper method of data collection by direct face-to-face interviews from Rabigh’s actual residents, adequate sample size and high power of the study (>95%), and in-depth systematic regression analyses with calculations of AOR have allowed for efficient estimation of the role of each risk factor in the development of childhood asthma.

The results of this survey with Identification of the main risk factors of childhood asthma should be useful in future targeted preventive plans/measures to limit the progressive alarming increase in the prevalence of asthma in children/adolescents, especially in Rabigh as well as in other industrial communities with similar conditions.

## 5. Limitations of the Study

This study has some limitations, including that the ISAAC study is based only on data collected from children and their families, and the accuracy of recall of some information may have been affected occasionally by the time span when asking about asthma/asthma symptoms events dating up to few years ago. The selection of the participants has been on a random basis, but the eastern and central areas of Rabigh are represented more in the study at 32.4% and 19.5% respectively when compared to western and southern areas, as the latter ones are mainly non-residential industrial zones. Objective measures have not been used to confirm the diagnosis of asthma carefully and precisely in the studied population. Additionally, respiratory viral infections known to induce wheezing have not been documented or identified. Therefore, the findings of this study should be interpreted with caution, but certainly, the prevalence rates of childhood asthma/asthma symptoms in Rabigh have strikingly increased compared to their previous recording more than 20 years ago. Moreover, the current 24-h fine particulate matter (PM2.5) indicative of air pollution has not been measured in Rabigh to confirm the ongoing poor air quality of Rabigh City, as documented in previous studies.

## 6. Conclusions

In this cross-sectional epidemiological survey, the prevalence rates of physician-diagnosed asthma, any wheezing, and wheezing in the last 12 months among children/adolescents (mean age: 12.22 ± 4.14 years) have remarkably increased from the previously recorded rates of 4.9%, 7.4%, and 6.4% in the only study that has been conducted in Rabigh in 1998 to 31.5%, 23.5%, and 14.9%, respectively, utilizing the same ISAAC questionnaire. Now, Rabigh can be considered to have the second highest prevalence of physician-diagnosed asthma of 31.5% in Saudi children, after Hofuf in the eastern region of KSA with a prevalence of 33.7%, and it is also higher than the reported general asthma prevalence among Saudi children from 8% to 25%, similarly recorded by the ISAAC questionnaire. The current rise in the prevalence of asthma/asthma symptoms in the industrial Rabigh City is associated with the significantly and rapidly developing industrialization, with many factories that have been established in the past 2 decades around Rabigh City. The role of all the demographic, socioeconomic, and biomedical variables has been examined and analyzed as risk factors for the development of childhood asthma/asthma symptoms. The univariate analysis has detected some significant risk factors of any wheezing, wheezing in the last 12 months, and physician-diagnosed asthma. However, only allergic rhinitis in children/adolescents and viral respiratory infection-induced wheezing remain as significant independent risk factors of any wheezing. Drug allergy in children/adolescents, exposure to dust, and viral respiratory infection induced-wheezing persist as the most significant independent risk factors of wheezing in the last 12 months, while eczema in the family, exposure to perfumes/incense, and viral respiratory infection induced-wheezing are the most significant independent risk factors of physician-diagnosed asthma in children/adolescents in Rabigh City.

## 7. Recommendations

The results of this survey and the identified main risk factors of childhood asthma should be useful in future targeted preventive plans/measures to limit the progressive and alarming increase in the prevalence of asthma in children/adolescents, especially in Rabigh as well as in other industrial communities with similar conditions. As the environmental risk factors are the major determinants of the prevalence of asthma in a community, special attention and management plans should concentrate on improving the poor air quality in Rabigh from TRAP and industry-related air pollution.

## Figures and Tables

**Table 1:** Sociodemographic characteristics of participants (*n* = 349).

Character		*n*	(%)
**Age: Mean, SD, Range**	12.22 ± 4.14 (5–18)		
**Children gender**	Male	144	41.3
	Female	205	58.7
**Residence area in Rabigh**	Eastern	113	32.4
	Western	34	9.7
	Central	68	19.5
	North	32	9.2
	South	30	8.6
	Northeast	29	8.3
	Northwest	12	3.4
	Southeast	19	5.4
	Southwest	12	3.4
**Residence near to factories**		82	23.5
**Factory type near residential area**	Petro Rabigh	41	11.7
	Cement	14	4.0
	Plastic	7	2.0
	Refining oil	6	1.7
	Concrete	2	0.6
	Steel	7	2.0
	Electricity/water	3	0.9
	Drinks	2	0.6
**Residence near main roads**		95	27.2
**Type of home**	Rent apartment	127	36.4
	Own apartment	103	29.5
	Rent villa	10	2.9
	Own villa	109	31.2
**Monthly family income**	Less than 3000 SAR	31	8.9
	3000–5000 SAR	77	22.1
	5000–10,000 SAR	107	30.7
	More than 10,000 SAR	134	38.4
**Father education**	Less than secondary school level	69	19.8
	Secondary school level	107	30.7
	University graduate	148	42.4
	Postgraduate studies	25	7.2
**Father job status**	Employed full time	273	78.2
	Self-employed/private business	45	12.9
	Unemployed	31	8.9
**Mother education**	Less than secondary school level	65	18.6
	Secondary school level	83	23.8
	University graduate	186	53.3
	Postgraduate studies	15	4.3
**Mother job status**	Employed full time	223	63.9
	Unemployed	126	36.1

**Table 2:** Prevalence of asthma/asthma symptoms according to ISAAC in children/adolescents in Rabigh city.

Asthma Symptoms		*n*	(%)
**Any wheeze anytime in the past**		82	23.5
**Wheezing in the last 12 months**		52	14.9
**Number of wheezing episodes in the last 12 months**	None	297	85.1
	1–3 times	43	12.3
	4–12 times	6	1.7
	More than 12 times	3	0.9
**Number of awakenings/disturbed sleep due to wheezing in the last 12 months**	None	310	88.8
	Once/week	34	9.7
	More than once/week	5	1.4
**Severe wheezing limiting speech in the last 12 months**		31	8.9
**Wheezing during or after exercise in the last 12 months**		35	10.0
**Dry nocturnal cough without colds in the last 12 months**		97	27.8
**Ever asthma/physician-diagnosed asthma**		110	31.5

**Table 3:** Multivariate regression analysis for risk factors of any wheezing in children and adolescents in Rabigh City.

Variable	Significance *p* Value	Adjusted Odds Ratio (AOR)	95% Confidence Interval for AOR	
			Lower Confidence Limit	Upper Confidence Limit
Age (children from 5–9 years)	0.04 *	0.48	0.24	0.95
Male gender	0.98	1.01	0.54	1.89
Residence near to main roads	0.46	1.30	0.650	2.59
Atopy in child/adolescent	0.86	0.89	0.24	3.24
Allergic rhinitis in child/adolescent	0.03 *	4.22	1.17	15.17
Allergic conjunctivitis in child/adolescent	0.99	1.0	0.22	4.46
Drug allergy in child/adolescent	0.86	0.89	0.24	3.25
Atopy in family	0.59	0.69	0.18	2.74
Atopy in mother	0.57	1.29	0.53	3.19
Atopy in sister	0.85	1.09	0.43	2.79
Allergic rhinitis in family	0.55	1.43	0.45	4.53
Eczema in family	0.43	1.45	0.59	3.57
Allergic conjunctivitis in family	0.62	1.35	0.41	4.43
Food allergy in the family	0.47	1.43	0.55	3.74
Drug allergy in the family	0.67	0.72	0.16	3.31
Carpet/Moquette in rooms	0.27	1.47	0.74	2.90
Exposure to passive smoking	0.09	1.89	0.90	3.99
Exposure to burned coal/wood	0.24	1.56	0.74	3.29
Exposure to perfumes/incense	0.67	0.82	0.34	2.01
Exposure to dust	0.99	1.00	0.47	2.09
Viral respiratory infection-induced wheeze	0.000 *	6.64	2.78	15.89
Associated chronic illnesses	0.007 *	5.52	1.59	19.16
Fast food more than twice/week	0.13	1.66	0.87	3.18

* Significance < 0.05.

**Table 4:** Multivariate regression analysis for risk factors of wheezing in the last 12 months in children and adolescents in Rabigh City.

Variable	Significance *p* Value	Adjusted Odds Ratio (AOR)	95% Confidence Interval for AOR	
			Lower Confidence Limit	Upper Confidence Limit
Age (children from 5–9 years)	0.96	1.00	0.88	1.15
Male gender	0.81	0.87	0.28	2.75
Residence near to main roads	0.27	1.86	0.62	5.58
Atopy in child/adolescent	0.84	1.23	0.17	8.69
Allergic rhinitis in child/adolescent	0.99	0.98	0.14	6.74
Eczema in child/adolescent	0.13	4.64	0.65	33.17
Allergic conjunctivitis in child/adolescent	0.18	5.70	0.45	72.958
Drug allergy in child/adolescent	0.01	11.34	1.66	77.48
Atopy in family	0.59	0.54	0.06	5.03
Atopy in mother	0.23	2.55	0.55	11.98
Atopy in brother	0.48	1.80	0.34	9.44
Atopy in sister	0.35	2.18	0.43	10.94
Allergic rhinitis in family	0.60	0.53	0.07	4.18
Eczema in family	0.65	0.70	0.16	3.16
Food allergy in the family	0.07	0.21	0.04	1.14
Exposure to passive smoking	0.27	0.47	0.12	1.80
Exposure to burned coal/wood	0.29	2.05	0.53	7.87
Exposure to perfumes/incense	0.29	0.41	0.08	2.12
Exposure to dust	0.03 *	3.82	1.10	13.24
Viral respiratory infections induced wheeze	0.01 *	4.63	1.33	16.05

* Significance < 0.05.

**Table 5:** Multivariate regression analysis for risk factors of physician-diagnosed asthma in children and adolescents in Rabigh City.

Variable	Significance *p* Value	Adjusted Odds Ratio (AOR)	Adjusted Odds Ratio (AOR)	
			Lower Confidence Limit	Upper Confidence Limit
Age (children from 5–9 years)	0.36	1.04	0.96	1.13
Male gender	0.74	0.89	0.48	1.68
Residence near to main roads	0.49	1.27	0.65	2.49
Atopy in child/adolescent	0.39	1.63	0.53	4.98
Allergic rhinitis in child/adolescent	0.13	0.39	0.12	1.29
Allergic conjunctivitis in child/adolescent	0.98	0.97	0.16	5.76
Drug allergy in child/adolescent	0.56	1.47	0.40	5.38
Atopy in family	0.79	1.20	0.31	4.67
Atopy in mother	0.47	1.39	0.57	3.37
Atopy in father	0.27	1.82	0.63	5.29
Atopy in brother	0.75	0.85	0.33	2.24
Atopy in sister	0.76	0.87	0.35	2.17
Allergic rhinitis in family	0.53	0.69	0.22	2.17
Eczema in family	0.03 *	2.85	1.10	7.41
Allergic conjunctivitis in family	0.06	3.48	0.97	12.54
Food allergy in the family	0.54	0.74	0.28	1.95
Moquette/carpet in rooms	0.67	0.86	0.44	1.69
Exposure to passive smoking	0.93	1.03	0.51	2.10
Exposure to burned coal/wood	0.48	1.30	0.63	2.71
Exposure to dust	0.17	1.71	0.79	3.68
Viral respiratory infections induced wheeze	0.001 *	6.35	2.24	18.02
Exposure to perfumes/incense	0.001 *	5.59	1.95	16.12

* Significance < 0.05.

## Data Availability

Data is contained within the article and Supplementary Material.

## Supplementary Materials

The following supporting information can be downloaded at: https://www.mdpi.com/article/10.3390/children10020247/s1, Table S1: Significant risk factors in univariate analysis which were associated with any wheeze in children and adolescents of Rabigh’s community; Table S2: Significant risk factors in univariate analysis which were associated with wheeze in the last 12 months in children and adolescents of Rabigh’s community; Table S3: Significant risk factors in univariate analysis which were associated with ever physician-diagnosed asthma in children and adolescents of Rabigh’s community.
