# Supplementary material for: Prevalence and Risk Factors of Asthma in Children and Adolescents in Rabigh, Western Saudi Arabia
Source: Children (Basel). 2023 Jan 30;10(2):247. doi: 10.3390/children10020247 (PMC9954887; doi:10.3390/children10020247)
Supplement: Supplementary file 1 [file children-10-00247-s001.zip › children-2146649-supplementary.pdf]

Table S1. Significant risk factors in univariate analysis which were associated with any wheeze in children and adolescents of Rabigh's community

Variable	Any wheeze (n=82) N	%	No wheeze (n=267) N	%	Odds ratio (95% CI)	X^2	P
**Residence near to main roads**	31	37.80	64	23.97	1.93 (1.14-3.27)	6.6	0.01
**Atopy in the child**	34	41.50	62	23.22	4.67 (2.76-7.88)	36.2	<0.0001
**Allergic rhinitis in the child**	43	52.43	43	16.10	5.74 (3.34-9.88)	44.6	<0.0001
**Allergic conjunctivitis in the child**	8	9.76	8	2.99	3.50 (1.27-9.64)	6.55	0.01
**Drug allergy in the child**	11	13.41	10	3.75	3.98 (1.63-9.75)	10.4	0.001
**Atopy in the family**	53	64.63	95	35.58	3.31 (1.97-5.55)	21.7	<0.0001
**Atopy in the mother**	28	34.15	46	17.23	2.49 (1.43-4.34)	10.7	0.001
**Atopy in the sister**	24	29.27	34	12.73	2.84 (1.56-5.15)	12.4	<0.0001
**Allergic rhinitis in the family**	42	51.22	75	28.09	2.69 (1.62- 4.47)	15.1	<0.0001
**Eczema in the family**	22	26.83	32	11.99	2.69 (1.46-4.97)	10.6	0.001
**Allergic conjunctivitis in the family**	13	15.85	12	4.49	4.00 (1.75-9.17)	12.2	<0.0001
**Food allergy in the family**	29	35.37	43	16.10	2.85 (1.63-4.98)	14.1	<0.0001
**Drug allergy in the family**	7	8.54	7	2.62	3.47 (1.18-10.19)	5.7	0.02
**Carpet/Mouquette in rooms**	53	64.63	127	47.57	2.01 (1.21-3.36)	7.32	0.007
**Exposure to passive smoking**	32	39.02	58	21.72	2.31 (1.36-3.92)	9.80	0.002
**Exposure to burned coal/wood**	25	30.49	48	17.98	2.00 (1.14-3.52)	5.69	0.02
**Exposure to perfumes/incense**	70	85.37	181	67.79	2.77 (1.43-5.38)	9.6	0.002
**Exposure to dust**	45	54.88	93	34.83	2.28 (1.38-3.76)	10.5	0.001
**Viral respiratory infection induced-wheeze**	25	30.49	14	5.24	7.93 (3.88-16.19)	40.2	<0.0001
**Fast food more than once/week**	39	47.56	76	28.46	2.28 (1.37-3.79)	10.4	0.001
**Associated chronic illness/comorbidity**	20	24.39	33	12.36	2.29 (1.23-4.26)	7.05	0.008

Table S2. Significant risk factors in univariate analysis which were associated with wheeze in the last 12 months in children and adolescents of Rabigh's community

Variable	Wheeze in the last 12 months (n=52)		No wheeze in the last 12 months (n=297)		Odds ratio (95% CI)	X^2	P
	N	%	N	%			
**Residence near to main roads**	23	44.23	72	24.24	2.48 (1.35-4.55)	8.9	0.003
**Atopy in the child**	34	65.38	75	25.25	6.09 (3.23 11.51)	36.3	<0.0001
**Allergic rhinitis in the child**	31	59.62	55	18.52	6.49 (3.47-12.15)	40.2	<0.0001
**Eczema in the child**	11	21.15	22	7.41	3.35 (1.51- 7.42)	9.77	0.002
**Allergic conjunctivitis in the child**	7	13.46	9	3.03	4.98 (1.77-14.03)	11.00	0.001
**Drug allergy in the child**	11	21.15	10	3.37	7.70 (3.08-19.26)	24.8	<0.0001
**Atopy in the family**	32	61.54	116	39.06	2.49 (1.36-4.57)	9.16	0.002
**Atopy in the mother**	18	34.62	56	18.86	2.28 (1.20-4.33)	6.58	0.01
**Atopy in the brother**	16	30.77	54	18.18	2.00 (1.04-3.86)	4.37	0.03
**Atopy in the sister**	16	30.77	42	14.14	2.69 (1.38-5.29)	8.83	0.003
**Allergic rhinitis in the family**	27	51.92	90	30.30	2.48 (1.37-4.52)	9.28	0.002
**Eczema in the family**	14	26.92	40	13.47	2.37 (1.18-4.75)	6.13	0.01
**Food allergy in the family**	17	32.69	55	18.52	2.14 (1.12-4.09)	5.40	0.02
**Exposure to passive smoking**	21	40.38	69	23.23	2.24 (1.21-4.14)	6.80	0.009
**Exposure to burned coal/wood**	17	32.69	56	18.86	2.09 (1.09-3.99)	5.12	0.02
**Exposure to perfumes/incense**	45	86.54	206	69.36	2.84 (1.23-6.54)	6.47	0.01
**Exposure to dust**	35	67.31	103	34.68	3.88 (2.07-7.26)	19.70	<0.0001
**Exposure to viral respiratory infections**	20	38.46	19	6.40	9.14 (4.42-18.91)	44.83	<0.0001

Table S3. Significant risk factors in univariate analysis which were associated with ever physician-diagnosed asthma in children and adolescents of Rabigh's community

Variable	Physician-diagnosed asthma (n=110)		No Physician-diagnosed asthma (n=239)		Odds ratio (95% CI)	X^2	P
	N	%	N	%			
**Residence near to main roads**	42	38.2	53	22.2	2.17 (1.32-3.54)	9.6	0.002
**Atopy in the child**	51	46.4	59	24.7	2.63 (1.64-4.25)	16.4	<0.0001
**Allergic rhinitis in the child**	42	38.2	44	18.4	2.73 (1.65-4.54)	16.3	<0.0001
**Allergic conjunctivitis in the child**	9	8.2	7	2.9	2.95 (1.07-8.15)	4.8	0.03
**Drug allergy in the child**	13	11.8	8	3.3	3.83 (1.54-9.53)	9.6	0.004
**Atopy in the family**	68	61.8	80	33.5	3.22 (2.01-5.14)	24.8	<0.0001
**Atopy in the mother**	34	30.9	40	16.7	2.23(1.31-3.77)	9.10	0.003
**Atopy in the father**	23	20.9	25	10.5	2.26 (1.22-4.20)	6.90	0.008
**Atopy in the brother**	31	28.2	39	16.3	2.01(1.17-3.45)	6.60	0.01
**Atopy in the sister**	27	24.5	31	13.0	2.18 (1.23-3.88)	7.30	0.007
**Allergic rhinitis in the family**	53	48.2	64	26.8	2.54 (1.59- 4.07)	15.5	<0.0001
**Eczema in the family**	35	31.8	19	7.9	5.40 (2.92-10.01)	32.8	<0.0001
**Allergic conjunctivitis in the family**	17	15.5	8	3.3	5.28 (2.20-12.65)	16.6	<0.0001
**Food allergy in the family**	34	30.9	38	15.9	2.37 (1.39- 4.03)	10.7	0.001
**Exposure to passive smoking**	40	36.4	50	20.9	2.16 (1.31- 3.55)	9.90	0.002
**Carpet/mouquette in home**	71	64.5	111	46.4	2.09 (1.32- 3.35)	9.90	0.002
**Exposure to burned coal/wood**	33	30.0	40	16.7	2.13 (1.25- 3.62)	7.70	0.006
**Exposure to perfumes/incense**	102	92.7	149	62.3	7.70 (3.58-16.56)	34.4	<0.0001
**Exposure to dust**	67	60.9	71	29.7	3.69 (2.30- 5.92)	30.7	<0.0001
**Exposure to viral respiratory infections**	32	29.1	7	2.9	13.60 (5.77- 32.04)	52.4	<0.0001
